# Supplementary material for: Evaluation of the setup discrepancy between 6D ExacTrac and cone beam computed tomography in spine stereotactic body radiation therapy
Source: PLoS One. 2021 May 27;16(5):e0252234. doi: 10.1371/journal.pone.0252234 (PMC8158872; doi:10.1371/journal.pone.0252234)
Supplement: S1 Table — (DOCX) [file pone.0252234.s001.docx]

Evaluation of the setup discrepancy between BrainLAB 6D ExacTrac and cone-beam computed tomography on the image guide system of the Novalis-Tx for spine stereotactic body radiation therapy

Jaehyeon Park^1,2^, Ji Woon Yea^1,2^, Jae Won Park^1,2^, and Se An Oh^1,2*^

^1^Department of Radiation Oncology, Yeungnam University Medical Center, Daegu, South Korea

^2^Department of Radiation Oncology, Yeungnam University College of Medicine, Daegu, South Korea

**Supporting Information**

**S1 Table**

| Spinal region | Number of patient | Directions | Setup error for  6D ExacTrac | | Setup error for  3D CBCT | | 6D ExacTrac vs 3D CBCT | | |
| --- | --- | --- | --- | --- | --- | --- | --- | --- | --- |
|  |  |  |  |  |  |  | Difference | | p-value of paired t-test |
|  |  |  | RMS | SD | RMS | SD | RMS | SD |  |
| Cervical | N=11, n=43 | Translational |  |  |  |  |  |  |  |
|  |  | Lateral (x-axis) (mm) | 0.37 | 0.37 | 0.99 | 0.60 | 1.17 | 0.61 | <0.001 |
|  |  | Longitudinal (z-axis) (mm) | 0.32 | 0.31 | 0.81 | 0.59 | 1.01 | 0.54 | <0.001 |
|  |  | Vertical (y-axis) (mm) | 0.37 | 0.36 | 1.28 | 1.18 | 1.05 | 0.84 | 0.005 |
|  |  | Rotational |  |  |  |  |  |  |  |
|  |  | Pitch (x-axis) (°) | 0.38 | 0.36 | n/a | n/a | n/a | n/a | n/a |
|  |  | Roll (z-axis) (°) | 0.48 | 0.48 | n/a | n/a | n/a | n/a | n/a |
|  |  | Yaw (y-axis) (°) | 0.36 | 0.36 | 0.70 | 0.67 | 0.89 | 0.56 | 0.032 |
| Thoracic | N=41, n=141 | Translational |  |  |  |  |  |  |  |
|  |  | Lateral (x-axis) (mm) | 0.43 | 0.43 | 1.12 | 0.87 | 1.34 | 0.83 | <0.001 |
|  |  | Longitudinal (z-axis) (mm) | 0.39 | 0.39 | 0.83 | 0.71 | 1.00 | 0.65 | <0.001 |
|  |  | Vertical (y-axis) (mm) | 0.34 | 0.34 | 0.87 | 0.87 | 0.86 | 0.56 | 0.097 |
|  |  | Rotational |  |  |  |  |  |  |  |
|  |  | Pitch (x-axis) (°) | 0.23 | 0.23 | n/a | n/a | n/a | n/a | n/a |
|  |  | Roll (z-axis) (°) | 0.29 | 0.29 | n/a | n/a | n/a | n/a | n/a |
|  |  | Yaw (y-axis) (°) | 0.31 | 0.31 | 0.42 | 0.41 | 0.62 | 0.43 | 0.026 |
| Lumbar | N=24, n=84 | Translational |  |  |  |  |  |  |  |
|  |  | Lateral (x-axis) (mm) | 0.37 | 0.37 | 1.11 | 0.79 | 1.32 | 0.82 | <0.001 |
|  |  | Longitudinal (z-axis) (mm) | 0.32 | 0.32 | 1.02 | 0.92 | 1.16 | 0.88 | 0.001 |
|  |  | Vertical (y-axis) (mm) | 0.27 | 0.27 | 0.88 | 0.88 | 0.79 | 0.57 | 0.245 |
|  |  | Rotational |  |  |  |  |  |  |  |
|  |  | Pitch (x-axis) (°) | 0.15 | 0.14 | n/a | n/a | n/a | n/a | n/a |
|  |  | Roll (z-axis) (°) | 0.22 | 0.22 | n/a | n/a | n/a | n/a | n/a |
|  |  | Yaw (z-axis) (°) | 0.24 | 0.24 | 0.34 | 0.34 | 0.52 | 0.34 | 0.588 |

^a^p<0.05. N, number of patients; n, number of fractions; RMS, root mean square; SD, standard deviation.

Residual setup errors between 6D ExacTrac and 3D cone-beam computed tomography (CBCT) for cervical, thoracic, and lumbar spines.
