# Supplementary material for: Evaluation of the setup discrepancy between 6D ExacTrac and cone beam computed tomography in spine stereotactic body radiation therapy
Source: PLoS One. 2021 May 27;16(5):e0252234. doi: 10.1371/journal.pone.0252234 (PMC8158872; doi:10.1371/journal.pone.0252234)
Supplement: Attachment — Submitted filename: 2-1.Response_to_Reviewers_ExacTrac vs CBCT.docx [file pone.0252234.s002.docx]

| **PONE-D-21-03969** | |
| --- | --- |
| **Reviewer #1** | **Revisions** |
| 1. Vac-lok fixtures refers to just a cradle and not BodyFix, correct? From the very small picture, it looks like it was just a regular Vac-lok. Please clarify. | Thank you for the comment. You are correct, we just used the regular Vac-lok. We have made the following revisions.  Abstract: A Vac-lok (EZ-FIX^®^, Arlico Medical Company, South Korea) fixture and an assistive device, based on the region involved, were used to immobilize patients and to increase the inter-fractional setup reproducibility.  Methods: For all patients, Vac-lok (EZ-FIX^®^, Arlico Medical Company, South Korea) fixtures were used to minimize patient movement and to increase interfractional setup reproducibility. |
| 2. I presume that an Exac-Trac registration was done followed by a CBCT registration and the final shifts were based on CBCT. Please clarify. | Thank you for the comment. We have made the following changes to clarify:  Finally, after the radiation oncologist confirmed the patient’s setup position, it was finally corrected using the 3D CBCT and radiotherapy was performed  (Image registration and setup protocol in Methods). |
| 3. Table 2- Please fix spacing of the table. | Thank you for the comment. The spacing in Table 2 has been adjusted. |
| **Reviewer #2** | **Revisions** |
| 1. Manuscript is sound and presents valuable data that is very relevant to this widespread technology.  It may be beneficial to include what margins were used for PTV expansion and whether the magnitude of uncertainty in the setup error would have any clinically significant effects on the dosimetry of the spine SBRT plan (PTV coverage, dose to OAR's, etc). Additionally, what factors are contributing to the residual setup discrepancy? Potential patient movement between ExacTrac and CBCT was mentioned...are there any other potential reasons (insufficient immobilization, anatomical motion, differences in image quality, etc)?  Do these factors differ between cervical, thoracic and lumbar spine regions? | Thank you for the comment. Information on the PTV expansion has been added.  The following sentences were added the in Methods (red-colored sentence):  In the case of spine SBRT with our institution protocol, planning target volume (PTV) was created by extending the margin by 3 mm in all directions from the clinical target volume. When major organs such as the spinal cord were adjacent, the PTV was reduced by the radiation oncologist.  The following sentences were modified in the Discussion (red-colored sentence):  In addition, Chang et al. suggested that the following are possible sources of residual setup discrepancy between ExacTrac and CBCT: (1) inter-scan patient motion, (2) the difference in image fusion algorithm, (3) poorer visibility of anatomical structures in digitally reconstructed radiographs and X-ray images, and (4) discrepancy of geometric accuracy for the two systems.    Unfortunately, this study did not consider residual setup discrepancy between ExacTrac and CBCT for cervical, thoracic, and lumbar spine regions. In the future, we will perform a study to find the cause of this difference depending on the regions between the cervical, thoracic, and lumbar spine regions. |
